# Supplementary material for: Predicting visual function by interpreting a neuronal wiring diagram
Source: Nature. 2024 Oct 2;634(8032):113–23. doi: 10.1038/s41586-024-07953-5 (PMC11446822; doi:10.1038/s41586-024-07953-5)
Supplement: Supplementary file 6 — CRF and ERF predictions for individual TmY4 and TmY9 cells. Analogous to Supplementary Data 3, but for TmY target types. Shown are the top four monosynaptic pathways, the strongest pathway passing through each of the top ten intermediary types (ranking from Extended Data Fig. 7), and the trisynaptic pathway Tm1–TmY–Dm3–TmY (see the section entitled Prediction of spatial normalization). [file 41586_2024_7953_MOESM6_ESM.zip › DataS4/TmY9q/720575940626502568.pdf]

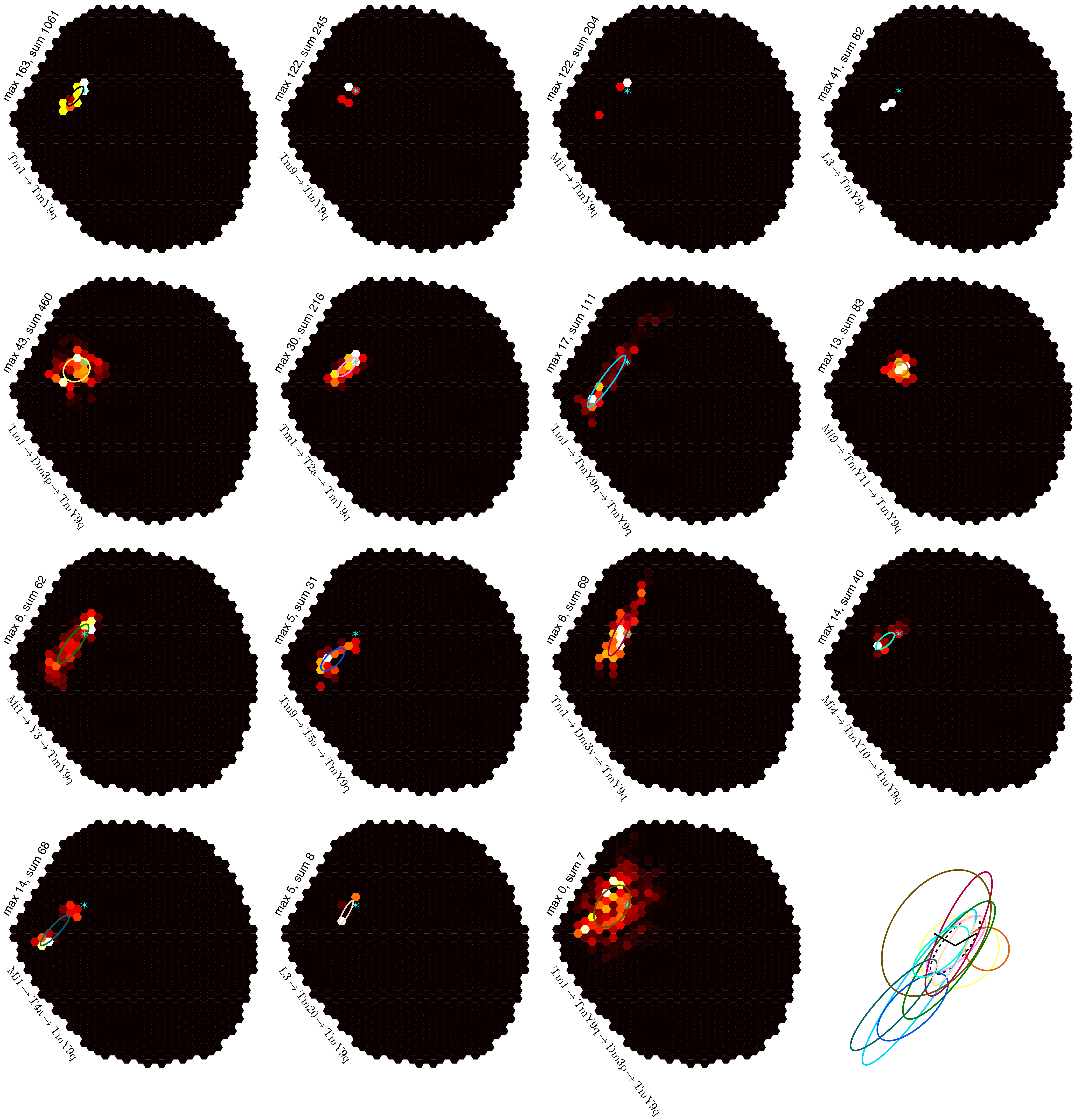

max 163, sum 1061
Tm1 → TmY9q
max 122, sum 245
Tm9 → TmY9q
max 122, sum 204
Mi1 → TmY9q
max 41, sum 82
L3 → TmY9q
max 43, sum 460
Tm1 → Dm3p → TmY9q
max 30, sum 216
Tm1 → T2a → TmY9q
max 17, sum 111
Tm1 → TmY9q → TmY9q
max 13, sum 83
Mi9 → TmY11 → TmY9q
max 6, sum 62
Mi1 → Y3 → TmY9q
max 5, sum 31
Tm9 → T5a → TmY9q
max 6, sum 69
Tm1 → Dm3v → TmY9q
max 14, sum 40
Mi4 → TmY10 → TmY9q
max 14, sum 68
Mi1 → T4a → TmY9q
max 5, sum 8
L3 → Tm20 → TmY9q
max 0, sum 7
Tm1 → TmY9q → Dm3p → TmY9q
